# ‘I can see that it’s bad for them’: third party judgements about the effect of procedural injustice on mental health and relationships with the police

**DOI:** 10.1007/s11292-025-09667-8

**Published:** 2025-02-25

**Authors:** Arabella Kyprianides, Ben Bradford

**Affiliations:** Department of Security and Crime Science, University College London, Shropshire House, 1 Capper Street, London, WC1E 6JA UK

**Keywords:** Exclusion, Identification, Legitimacy, Mental health, Procedural injustice, Trust

## Abstract

**Objectives:**

Explore why people react so strongly to procedural *injustice* experienced by others. One possibility is that people recognise the marginalisation and psychological harm that injustice can cause.

**Methods:**

An online experiment tested whether exposure to procedurally unjust police behaviour directed at crime victims would influence participants’ assessments of the subject’s marginalisation, mental health outcomes, and their overall perceptions of the police (*N* = 300).

**Results:**

Exposure to procedurally unjust police behaviour led to more negative evaluations of its psychological impact on the victim. Procedural injustice was seen as harmful to mental health due to its link to social exclusion. Such exposure reduced trust, legitimacy, and identification with police. These negative perceptions were driven by recognition of the victim’s psychological harm.

**Conclusion:**

Findings highlight the critical role of psychological harm in driving public responses to unjust policing. Addressing this harm is essential for fostering trust and repairing fractured relationships between police and the communities they serve.

## Introduction

The way people perceive police behaviour can have significant implications for both public trust in police and individuals’ mental well-being. Research has demonstrated that direct and vicarious experiences with policing can negatively impact individuals’ mental health (McLeod et al., 2020; Jindal et al., 2022) and shape their attitudes toward the police (Bolger & Walters, 2019; Tyler, 2011; Walters & Bolger, 2019). However, while we know that exposure to policing can erode trust and increase psychological distress, less is understood about the underlying psychological mechanisms driving these responses. Specifically, less is known about why people seem to react so strongly to procedural injustice,^1^ even in situations where they are not themselves directly involved (Foster et al., 2022; Johnson et al., 2017; Ray, 2023; Williamson & Murphy, 2023).

One potential explanation is that people’s understanding of the impact of injustice *on others* plays a crucial role in shaping their reactions. Recognising the exclusion and emotional harm inflicted on victims of procedural injustice may evoke empathy or moral outrage (Darley, 2009), amplifying the intensity of their responses. When people feel that moral norms—such as the expectation that powerholders treat subordinates with procedural justice (Mackenzie, 2020; Tyler & Bies, 2015)—have been violated, they can respond with anger and seek retribution against the transgressor, even when they themselves are not directly involved. As Miller (2001, cited in Darley, 2009) notes:

The arousal of moralistic anger is not confined to injustices perpetrated against one’s self. Witnessing the harming of a third party can also arouse strong feelings of anger and injustice. … Individuals are committed to the “ought forces” of their moral community, as Heider (1958) termed them, and people believe that these forces deserve respect from all members of the community. The violation of these forces represents an insult to the integrity of the community and provokes both moralistic anger and the urge to punish the offender ...

This suggests that perceptions of exclusion and psychological harm are plausible elements of the broader mechanisms through which police procedural injustice influences public attitudes of the police. When normative expectations are disappointed and, in particular, when another is perceived to be suffering from the action or inaction of a third party, trust in that third party may be diminished (Walker, 2006). This study investigates why people perceive unfairness as problematic in the context of police interactions with crime victims. We explore how perceptions of police procedural justice shape peoples’ judgements of these interactions: specifically, how evaluations of procedural justice influence assessments of the victim’s marginalisation and mental health, as well as general perceptions of the police, including trust, legitimacy, and identification. Using an experimental design with a text-based vignette, we manipulate police behaviour to evaluate how police procedural justice influences reactions to these vicarious encounters. Whereas much of the procedural justice literature focuses on police interactions with suspects or offenders, this study considers the public’s perception of procedural injustice directed toward crime victims, who are generally viewed as more deserving of fair and supportive treatment (Charman & Williams, 2022; Williamson & Murphy, 2023).

Our study thus investigates the public’s recognition of the emotional and social harm caused by procedurally unjust police behaviour and its impact on perceptions of the police. Specifically, we aim to address the following four research questions:

1. Emotional and social harm awareness:

Do individuals recognise the emotional harm caused by police procedural injustice to others and understand that it can contribute to marginalisation and social exclusion?

2. Role of procedural justice in perceptions of harm:

Do perceptions of procedural justice influence judgements about the mental health impact of police behaviour through an awareness of the exclusion experienced by others?

3. Impact on general perceptions of the police:

Does vicarious exposure to procedurally unjust police behaviour damage general perceptions of the police, including trust, legitimacy, and identification?

4. Mediation by exclusion and psychological harm:

Are negative perceptions of the police influenced by an awareness of the exclusion and psychological harm experienced by others?

By addressing these questions, this study enhances our understanding of lay judgements about the effect of unfair policing. It also explores the broader effects of unjust and exclusionary police behaviour on public perceptions, while uncovering what drives these responses.

### The effects of police behaviour on mental health

Research has demonstrated that direct and vicarious experiences with policing can negatively impact individuals’ mental health (McLeod et al., 2020; Jindal et al., 2022). In a systematic review, McLeod et al. (2020) examined how police encounters impact the mental health of Black Americans. The review covered a wide range of interactions, from the use of force during arrests to routine stops, searches, witnessing police violence, and encounters within the judicial system. The mental health outcomes included conditions such as psychosis, psychological distress, depression, PTSD, anxiety, and suicidal thoughts or attempts. Of the eleven studies reviewed, six found statistically significant links between police interactions and mental health issues, with individuals who had experienced police encounters nearly twice as likely to report poor mental health compared to those without such experiences.

In another systematic review, Jindal et al. (2022) focused on the effects of police exposure on Black youth aged 26 and younger. This review analysed 16 quantitative studies involving 19,493 participants, finding consistent associations between police encounters and negative outcomes such as mental health deterioration, increased sexual risk behaviours, and substance use. Additionally, 13 qualitative studies, including 461 participants, supported these findings and revealed other concerns, such as increased fear for personal safety and feelings of hopelessness. While neither systematic review specifically addressed interactions between police and crime victims, existing literature has documented comparable negative mental health outcomes resulting from such interactions (Brooks-Hay, 2020; Hohl et al., 2023; Jordan, 2004; Hohl & Stanko, 2024; Greeson et al., 2014).

Although we know that exposure to policing can negatively affect mental health, the psychological mechanisms that contribute to these responses remain less understood. In other words, what specific aspects of policing are responsible for driving these negative outcomes?

#### Police procedural injustice and exclusion

An emerging idea suggests that the psychological impact of police encounters depends on how people construe them (Alang et al., 2021). Although still in the early stages of development, this perspective indicates that perceptions of fair police behaviour can enhance mental well-being, while perceptions of injustice may lead to negative psychological outcomes.

In this context, theories of police-community relations are valuable for understanding how individuals assess their interactions with police. The leading framework in this area is procedural justice theory (PJT), which highlights the importance of fair and respectful treatment by officers in shaping how both individuals and communities perceive and respond to the police (Walters & Bolger, 2019). PJT focuses on the fairness of how authorities, such as the police, exercise their power, and is built around four key elements: allowing individuals to voice their concerns, ensuring decisions are impartial, treating people with dignity and respect, and providing clear, transparent processes.

According to PJT, when individuals perceive that the police have treated them fairly, they are more likely to trust the police and view their actions as legitimate. This perception of fairness also encourages stronger identification with the social categories the police represent, such as the law-abiding public or the community at large (Chan et al., 2024; Walters & Bolger, 2019). While research has shown that procedural justice behaviours significantly shape how communities perceive the police (Walters & Bolger, 2019), their role in directly influencing mental health outcomes remains underexplored.

Three notable studies provide some insight. Geller et al. (2014), using US population survey data, found that young men who experienced police stops and perceived the interactions as fair, ethical, and respectful reported fewer symptoms of anxiety and PTSD compared to those who viewed the stops as unjust. Building on this, Alang et al. (2021) used larger national samples to show that police use of force perceived as unjust had a more harmful effect on mental health than force perceived as justified. Most recently, Gearhart et al. (2023) highlighted the protective role of procedural justice in mitigating post-traumatic stress among youths, showing that fair and respectful verbal interactions by officers helped reduce the adverse psychological effects of police encounters. Mcfarland et al. (2019) suggest that these effects arise because the experience of unjust police practice may lead people to ‘perceive the situation as unpredictable, highly threatening, and difficult to navigate – the characteristics that make stressors stressful (Sapolsky, 2004)’. Additional support for this idea comes from qualitative studies that explore the personal experiences of individuals who have encountered or heard about perceived unjust policing practices. For instance, Brunson and Weitzer (2009), in interviews with male adolescents from a US city, found that factors like physical abuse, perceived corruption, unwarranted stops, and verbal abuse by officers contributed to a sense of hopelessness among the participants.

Research from outside the field of policing highlights the importance of procedural justice in promoting psychological well-being. In workplace studies, perceived procedural justice has been consistently linked to better employee mental health, stress-related health problems, and lower levels of sick absence (Cachon-Alonso & Elovainio, 2022). For example, when employees feel valued and respected, their motivation and morale increase, and they are less likely to experience feelings of resentment or disengagement (Cachon-Alonso & Elovainio, 2022), and involving employees in decision-making processes has been shown to reduce stress levels (Brotheridge and Lee, 2003). These positive emotional states lead to a reduction in conflicts and grievances (Cropanzano et al., 2007; Kerwin et al., 2015; Lee, 2018), greater trust in the organisation (Cropanzano et al., 2007; Lee, 2018; Mukherjee and Bhattacharya, 2013; Smith & Lindsay, 2014; Williamson & Williams, 2011), and higher commitment and loyalty to the company (Cropanzano et al., 2007; González-Cánovas et al., 2024; Mukherjee and Bhattacharya, 2013).

In organisational justice research, high levels of procedural justice have also been found to buffer the negative effects of low distributive justice (i.e., unfair distribution of rewards or risks). Fields et al. (2000) found that while low levels of both procedural and distributive justice were linked to the lowest job satisfaction, high procedural justice helped reduce the negative impact of low distributive justice on job satisfaction (also see Brockner & Wiesenfeld, 2005; Vermunt & Steensma, 2003; Versteegt et al., 2022).

The concept of inclusionary police conduct is closely tied to procedural justice, particularly in the context of police-victim interactions. In these encounters, police officers often act as representatives of the state or society, bearing the responsibility to address victims’ concerns and ensure their voices are heard within the larger framework of justice. Procedural justice in these interactions goes beyond enforcing the law; it involves treating victims with dignity, respect, and impartiality, all of which contribute to their perception of being valued by the system. When victims feel their experiences are dismissed, trivialised, or not taken seriously, it reinforces feelings of alienation, exclusion, and marginalisation. This exclusion is not just personal but symbolic, reflecting a broader sense of being sidelined by systems of power and justice that are supposed to protect. Such experiences are extensively documented in the victim literature, which highlights the significant role police behaviour plays in shaping victims’ psychological and emotional outcomes (Brooks-Hay, 2020; Hohl et al., 2023). Dismissive or exclusionary policing undermines victims’ trust in the justice system and society as a whole, further exacerbating their feelings of vulnerability and disconnection.

Victims who perceive unfair treatment or procedural injustice in their interactions with police frequently report withdrawing from engagement with both police and broader support systems. This withdrawal often stems from a sense of isolation, disempowerment, and even betrayal by the very institutions they expected to support them (Hohl & Stanko, 2024; Jordan, 2004). Inefficient, dismissive, or indifferent police responses convey a message that victims are unworthy of care, respect, or recognition, reinforcing negative self-perceptions and societal stigma. This lack of inclusion can impact victims’ mental health, contributing to heightened stress, anxiety, and feelings of helplessness. On the other hand, when police behaviour communicates care, acknowledgment, and a genuine commitment to justice, it can affect victims in positive ways. Being treated fairly and with respect fosters a sense of validation, worthiness, and support, which can help mitigate the psychological harm of the victimisation experience itself (Greeson et al., 2014; Hohl et al., 2023).

In short, there is much to suggest that procedural injustice can be bad for people’s mental and physical health. In this paper, though, we consider a slightly different question, which is whether people recognise that procedural injustice is bad for *other* people’s health. We do so for two reasons. First, there is much evidence to suggest that vicarious contact with police can, when judged unfair, damage perceptions of procedural justice, trust, legitimacy, and other outcomes in much the same way as direct, personal contact does (Foster et al., 2022; Johnson et al., 2017; Ray, 2023; Williamson & Murphy, 2023). Vicarious encounters with police can provide people with important information—that the police are trustworthy or not, for example—upon which to base their judgements. Less clear, perhaps, is what people think the impact of procedural (in)justice is on others. Most classic accounts of procedural justice stress that it is important for people because it reduces uncertainty, provides a sense of control, and (re)affirms group membership (Thibaut & Walker, 1975; van den Bos & Lind, 2002; de Cremer & Blader, 2006). These are all centred on the individual, or, rather, the individual’s relationship with police. But do people recognise the psychological harm that procedural injustice can do to others, and do they see that it can serve to marginalise and exclude? If they do, this might help further explain why vicarious experiences of policing damage trust in the police and police legitimacy.

### Does police behaviour affect public opinion due to the recognition of psychological harm?

In our study, we examine (a) whether individuals recognise the emotional harm police procedural injustice causes others and its role in fostering marginalisation and social exclusion [RQ1] and (b) whether perceptions of procedural justice influence judgements about the mental health impact of police behaviour through an awareness of the exclusion experienced by others [RQ2]. Our analyses take socio-demographic factors into account because age, gender, ethnicity, and socio-economic status can influence perceptions of police behaviour (Bradford et al., 2017; Hu et al., 2020), and prior experiences with victimisation and social vulnerability may also affect views on procedural justice (Koster et al., 2018).

As we have already seen, a substantial body of literature documents the significant costs associated with procedurally unjust policing. Trust, legitimacy, and police identification are central to the PJT literature and are positioned as three important outcomes of procedurally fair treatment. These patterns have been observed in the context of both direct experiences with policing and vicarious experiences (Bolger & Walters, 2019; Tyler, 2011; Walters & Bolger, 2019). We therefore examine whether reading about procedurally unjust police behaviour toward a crime victim affects the public’s trust in the police, perceptions of police legitimacy, and levels of identification with the police [RQ3], but we also consider whether any negative impact on people’s general perceptions of the police is due to their recognition of the exclusion experienced by the victim or the psychological harm inflicted on the victim’s mental health [RQ4]. Understanding this mediation process is important because it could reveal that it is not merely the unfair actions themselves, but the perceived marginalisation and harm to victims, that amplifies distrust and disconnection from police.

While it is well established that vicarious exposure to unjust police actions—where observers witness or learn about the mistreatment of others—can erode trust in the police, the notion that recognising feelings of exclusion and psychological harm mediates the relationship between unjust police practices and broader evaluations of police trust, legitimacy, and identification remains unexplored. However, there is evidence to support our proposition that comes from research highlighting people’s sensitivity to how authorities treat vulnerable individuals, particularly crime victims.

Crime victims are generally perceived especially deserving of fair, compassionate, and dignified treatment, particularly by authorities like the police, who are entrusted with upholding justice and protecting vulnerable members of society (c.f. Williamson & Murphy, 2023). When police fail to treat victims with the expected care, people are likely to respond with strong disapproval. This reaction is likely to be intensified when the police inflict feelings of exclusion or psychological harm on crime victims, as it might be seen as a serious violation of social expectations regarding how the vulnerable should be treated.

Recognising the potential psychological harm done to crime victims during interactions with police could be an important factor that shapes how people evaluate the police more broadly.

When police practices are perceived as unjust, particularly if they result in feelings of exclusion or mental distress for victims, it highlights a fundamental failure to fulfil the police’s societal role. This failure not only reflects poorly on the individual officers involved but also taints public perceptions of the institution as a whole. By acknowledging the psychological damage done to victims, observers might personalise the failure to ‘this officer’ or interpret such incidents as emblematic of systemic issues, reinforcing the belief that police practices are exclusionary and unfair.

The recognition of psychological harm may thus serve as a mediator between unjust police conduct and the erosion of trust, legitimacy, and identification with the police. When citizens perceive that officers disregard the well-being of those already suffering, it undermines trust in the police as protectors of the community. In parallel, the police are no longer seen as legitimate representatives of justice but rather as an ‘out-group’ that operates contrary to the values of fairness, compassion, and integrity that the community expects. This perceived disconnection from shared moral principles ultimately diminishes the willingness of citizens to identify with and support the police.

We therefore examine whether reading about unjust police actions is connected to recognising the psychological harm they cause—such as feelings of exclusion and mental health impacts—and how these perceptions affect trust, legitimacy, and emotional attachment to the police.

## The current research

We conducted an online text-based vignette experiment to answer our research questions relating to whether exposure to procedurally just or unjust police behaviour would influence participants’ assessment of the mental health consequences of the interaction and their perceptions of the police. The text-based vignette described an interaction between two police officers and a crime victim who had called for assistance after being mugged. We manipulated the officers’ behaviour to reflect either procedurally just or procedurally unjust conduct. Although the vignettes present a hypothetical scenario, previous research has shown that varying officer behaviour through text-based vignettes can successfully shift participants’ judgements of, for example, police legitimacy (e.g. Silver and Pickett, 2015) and trust (Kyprianides et al., 2021). We specify four hypotheses to answer our research questions:

- H1. When exposed to a scenario of procedurally unjust police behaviour, people will evaluate the police-victim interaction, and the psychological harm inflicted on the victim (exclusion and emotional harm) more negatively [RQ1].
- H2. Higher perceptions of procedural justice in police interactions will lead to more favourable judgements about the victim’s mental health by reducing perceptions of the victim’s social exclusion [RQ2].
- H3. Exposure to a scenario describing procedurally unjust police behaviour will result in a loss of trust, legitimacy, and identification with the police [RQ3].
- H4. Perceptions of unfair police practices will influence participants’ views of the police through their perceptions of the victim’s exclusion and the harm caused to the victim’s mental health [RQ4].

### Method

#### Participant information

The study was hosted on Qualtrics. UK residents were recruited via the online crowdsourcing platform Prolific. In line with Prolific recruitment protocols, participants received compensation for their time. We followed Chandler and Paolacci’s (2017) advice on how to minimise participant fraud on Prolific: we set constraints so that participants could only take the survey once and included attention checks throughout the surveys. Participants were excluded if they got more than one attention check wrong. The final sample comprised 300 participants, representative of the UK adult population (see Table 1). For instance, the distribution of gender, ethnicity, and age in our sample closely aligns with official UK population statistics (ONS, 2021).

**Table 1:** Participant information

Characteristic	Category^*^	%	N	PJ condition % *N*	P unjust condition % *N*
Gender	Male	47.8	144	51.0 77	44.7 67
	Female	51.2	154	48.3 73	54.0 81
	Other	0.3	3	0.7 1	1.3 2
Age range	18–24	10.6	32	11.9 18	9.3 14
	25–44	32.6	98	17.9 27	15.3 23
	45–64	39.2	118	16.6 25	15.3 23
	65 +	17.6	53	16.6 25	18.0 27
Ethnicity	White	82.1	247	76.2 115	82.7 124
	Asian	4.0	12	4.6 7	4.7 7
	Black	4.0	12	2.6 4	3.3 5
	Mixed	3.3	10	6.0 9	4.0 6
	Other	3.0	9	6.2 9	5.3 8
Sexual orientation	Heterosexual	87.4	263	88.7 134	86.0 129
	Other	12.6	38	11.3 17	14.0 21
Education	Lower-level qualification	29.9	90	29.9 43	30.0 45
	Mid-level qualifications	16.3	49	17.9 27	18.0 27
	Higher level qualifications	57.5	173	53.7 81	52.0 78
Employment status	Active employment/ work	60.8	183	56.3 85	52.0 78
	Non-working but engaged in other activities	28.2	85	28.2 45	30.7 46
	Out of work or unable to work	11.0	33	15.5 21	17.3 26
Disability	Yes	23.9	72	25.2 38	22.7 34
	No	76.1	229	74.8 113	77.3 116
Residential area	Urban	26.6	80	25.2 38	28.0 42
	Suburban	52.8	159	54.3 82	51.3 77
	Rural	20.6	62	20.5 31	20.7 31
Deprivation	Financially stable	65.4	197	65.4 95	65.3 98
	Financially struggling	34.6	104	34.6 56	34.7 52
Financial difficulty	Easy	46.5	140	46.3 70	47.3 71
	Neutral	23.9	72	24.5 37	24.0 36
	Difficult	29.6	89	29.2 44	28.7 43
Victimisation history	Yes	23.3	70	20.5 31	26.0 39
	No	76.7	231	79.5 120	74.0 111

*Note that the categories have been consolidated for simplicity of presentation

## Experimental procedure

Participants were first asked about their victimisation experiences, serving as a useful prompt for them to think about what it is like to be a victim of crime. Participants were then randomly exposed to one of two short, text-based vignettes depicting a police interaction with a victim of a crime. The vignettes varied in terms of procedural justice or injustice (see Appendix 1). Participants were then asked questions about their judgements of these interactions, their implications, and their perceptions of the victim’s mental health outcomes, specifically: judgements of procedural justice/injustice displayed by the police; perceptions of inclusion/exclusion experienced by the victim during the interaction; and perceptions of the victim's mental health outcomes (e.g. stress levels, emotional well-being) as a result of the interaction. Participants were then asked questions on their general perceptions of the police, including perceptions of police procedural justice, perceptions of police legitimacy, and levels of social identification with the police. Lastly, participants provided responses to a series of demographic and socio-economic questions.

## Measures

Participants responded to a series of measures, mostly using 5-point scales, with the exception of demographic and socio-economic variables. See Appendix 2 for a list of the items used and the scale reliability for all measures (all *α* > 0.8).

To assess judgements of procedural justice or injustice displayed by the police, participants rated overall fair treatment, service satisfaction, and case satisfaction, each on single-item scales. Additionally, a procedural justice scale consisting of 9 items was used to capture more nuanced perceptions of fairness. Perceptions of inclusion or exclusion experienced by the victim were measured using a 4-item scale focused on exclusion. Perceptions of the victim’s mental health outcomes, specifically stress levels and emotional well-being following the interaction, were measured using two scales: a 19-item scale assessing the victim’s mental health and another 19-item scale reflecting how participants believed their own mental health would fare in the victim’s position.

Participants’ broader perceptions of the police were measured across several domains. A 2-item scale measured trust in the police, a 6-item scale captured police legitimacy, and a 5-item scale measured participants’ identification with the police. In addition to these scales, demographic variables such as age, gender, sexual orientation, and ethnicity were recorded, along with socio-economic variables including education, employment status, disability, residential area, deprivation, financial difficulty, and victimisation history. A final qualitative question invited participants to provide any additional comments or feedback.^2^

## Results

**Fig. 1:**
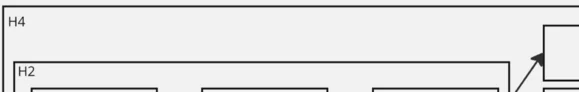
Conceptual framework for the path models

The analysis involves a combination of one-way ANOVAs and path modelling to test the study’s hypotheses. One-way ANOVAs will examine the effects of the experimental conditions (procedurally just vs. procedurally unjust) on participants’ judgements of the interaction and perceptions of psychological harm, operationalised as social exclusion and emotional harm [H1]. A path model will be specified with perceptions of procedural justice as the independent variable, perceptions of the victim’s mental health as the dependent variable, and perceptions of exclusion as the mediator. This model will assess both direct effects and the mediating role of exclusion [H2]. Three one-way ANOVAs will test the effects of the experimental conditions on trust in the police, perceptions of police legitimacy, and identification with the police [H3]. Three path models will be specified with perceptions of procedural justice as the independent variable, trust (Model 1), legitimacy (Model 2), and identification with the police (Model 3) as the dependent variables. Perceptions of exclusion and the victim’s mental health will be included as mediators to evaluate direct and mediated effects [H4]. Figure 1 illustrates the conceptual framework for the path models.

The models were evaluated using the PROCESS macro for SPSS, which estimates direct, indirect, and total effects in mediation models. To assess the significance of mediation effects, we used bootstrapping with 10,000 resamples to generate bias-corrected confidence intervals, ensuring robust estimation of indirect effects. Model evaluation also included reporting *R*^2^ values to assess the proportion of variance explained by the predictors. Additionally, the statistical significance (*p*-values) of path coefficients and the confidence intervals for mediation effects were examined.

### Manipulation checks

Manipulation checks were conducted to verify whether the vignettes effectively influenced participants’ perceptions of procedural justice. This was done by comparing ratings of procedural justice between participants in the procedurally just condition and those in the procedurally unjust condition. Participants in the procedurally just condition rated the police interaction as more procedurally fair (*M* = 4.62, SD = 0.51) compared to those in the procedurally unjust condition (*M* = 2.59, SD = 0.61). ANOVA results confirmed these differences, with procedural justice ratings showing a large effect size (*F*(1, 299) = 970.31, *p* < 0.001, *η*^2^ = 0.764).

### Main effects of police behaviour on participants’ judgements of the interaction and perceptions of psychological harm inflicted on the victim

First, we ran a series of one-way ANOVAs to assess the effects of the experimental conditions (procedurally just condition vs. procedurally unjust condition) on participants’ judgements of the interaction and perceptions of psychological harm inflicted on the victim in terms of social exclusion and emotional harm [H1]. The ANOVAs indicated significant differences between participants in the procedurally just condition and those in the procedurally unjust condition across all measures. Descriptive statistics are presented in Table 2.

**Table 2:** Descriptive statistics for participant judgements of the interaction and psychological harm inflicted on the victim by police behaviour condition

Judgement	Police behaviour condition				*F*(1, 299)	*η*^2^
	Procedurally just		Procedurally unjust			
	*M*	*SD*	*M*	*SD*		
Overall fair treatment (high = more)	4.76	0.57	2.21	0.82	974.77***	765
Service satisfaction (high = more)	4.70	0.61	1.65	0.72	1557.60***	0.839
Case satisfaction (high = more)	4.64	0.61	1.67	0.71	1509.07***	0.835
Exclusion (high = more)	1.48	0.50	3.25	0.82	505.09***	0.628
The victim’s mental health (high = better)	2.87	0.48	1.55	0.32	794.56***	0.727
Participants own mental health in victim’s position (high = better)	2.84	0.56	1.52	0.38	569.34***	0.656

***p < 0.001

#### Assessments of police-victim interaction

Those in the procedurally unjust condition thought that Emily was overall treated less fairly compared to those in the procedurally just condition (*M* = 2.21, SD = 0.82 vs. *M* = 4.76, SD = 0.57; *F*(1, 299) = 974.77, *p* < 0.001, *η*^2^ = 0.765). They also reported that they would have been less satisfied with the service they received if they were Emily (*M* = 1.65, SD = 0.72 vs. *M* = 4.70, SD = 0.61; *F*(1, 299) = 1557.60, *p* < 0.001, *η*^2^ = 0.839) and that they would have been less satisfied with how the police dealt with the case if they were Emily (*M* = 1.67, SD = 0.71 vs. *M* = 4.64, SD = 0.61; *F*(1, 299) = 1509.07, *p* < 0.001, *η*^2^ = 0.835), compared to those in the procedurally just condition.

#### Perceptions of the psychological harm inflicted on the victim

Participants in the procedurally unjust condition perceived the victim as more excluded (*M* = 3.25, SD = 0.82) compared to those in the procedurally just condition (*M* = 1.48, SD = 0.50). ANOVA results confirmed these differences, with exclusion ratings showing a large effect size (*F*(1, 299) = 505.09, *p* < 0.001, *η*^2^ = 0.628). Additionally, participants in the procedurally unjust condition believed that the interaction left Emily feeling more fearful, depressed, angry, helpless, isolated, and confused, while feeling less validated, hopeful, safe, trusting of authority, understood, and reassured compared to those in the procedurally just condition. They expressed similar sentiments when asked to imagine themselves in Emily’s position, considering how they would feel after the interaction with the police. In particular, participants in the unjust condition perceived Emily as having a less positive mental state following the interaction (*M* = 1.55, SD = 0.32 vs. *M* = 2.87, SD = 0.48; *F*(1, 299) = 794.56, *p* < 0.001, *η*^2^ = 0.727). Similarly, they believed they themselves would feel less positive if they were in Emily’s situation (*M* = 1.52, SD = 0.38 vs. *M* = 2.84, SD = 0.56; *F*(1, 299) = 569.34, *p* < 0.001, *η*^2^ = 0.656).

In line with H1, then, when exposed to police behaviour, people viewed the police-victim interaction, and the psychological harm inflicted on the victim (in terms of exclusion and emotional harm) more negatively when the police behaviour was unjust.

### The influence of procedural justice in shaping perceptions of psychological harm

Next, to test H2, we specified a path model with perceptions of procedural justice as the independent variable, perceptions of the victim’s mental health the dependent variable, and perceptions of exclusion as the mediator. Age, gender, sexual orientation, ethnicity, education, employment status, disability, residential area, deprivation, financial difficulty, and victimisation were entered as control variables. The model included all possible paths to assess (a) the direct effect of procedural justice on perceptions of exclusion and the victim’s mental health (see Table 3) and (b) the extent to which the effect of procedural justice on perceptions of the victim’s mental health is mediated by perceptions of exclusion (see Table 4), accounting for socio-demographic variables. The model explained 84% of the variance in perceptions of the victim’s mental health (*R*^2^ = 0.84).

**Table 3:** Perceptions of officer procedural justice predicting perceptions of victim exclusion and mental health

	Victim’s sense of exclusion		Victim’s mental health		
	B	SE	B	SE	
Procedural justice	− 0.80**	**0.03**	0.44**	**0.04**	
Exclusion			− 0.17**	**0.04**	
Age	0.01	0.02	− 0.04*	0.01	
Gender	− 0.12	0.07	0.02	0.04	
Sexual orientation	0.01	0.03	0.01	0.02	
Ethnicity	− 0.01	0.01	0.01	0.01	
Education	0.01	0.02	− 0.03*	0.01	
Employment	0.01	0.02	0.01	0.01	
Disability	0.06	0.09	0.04	0.06	
Residential area	− 0.06	0.06	− 0.05	0.04	
Deprivation	0.03	0.05	− 0.01	0.03	
Financial difficulty	− 0.02	0.04	− 0.03	0.02	
Victimisation	− 0.08	0.09	0.14*	0.06	

**p < 0.001, *p < 0.05

**Table 4:** Mediation analysis summary

Relationship	Total effect	Direct effect	Indirect effect	CI		Conclusion
				Lower	Upper	
PJ > Exclusion > MH	0.58 (*p* < 0.001)	0.44 (*p* < 0.001)	0.21	0.084	0.319	Partial mediation

The direct effect of perceptions of procedural justice on perceptions of exclusion was significant (*B* =  − 0.80, SE = 0.03, *p* < 0.001). The direct effect of perceptions of procedural justice on perceptions of the victim’s mental health in the presence of this mediator (perceptions of exclusion) was also significant (*B* = 0.44, SE = 0.04, *p* < 0.001). The indirect effect of perceptions of procedural justice on perceptions of the victim’s mental health via perceptions of exclusion was also significant (indirect effect = 0.21 [0.08, 0.32]). This suggests that perceptions of exclusion partially mediated the relationship between perceptions of procedural justice and perceptions of the victim’s mental health.

In other words, participants who perceived procedural justice were more likely assess the victim’s mental health positively, partly because, it seems, they felt police procedural justice reduced feelings of social exclusion experienced by the victim. Importantly, these relationships remained robust even after controlling for a range of socio-demographic factors, including participants’ age, gender, sexual orientation, ethnicity, education, employment status, disability, residential area, level of deprivation, financial difficulty, and prior victimisation.

### Main effects of police behaviour on participants’ perceptions of the police

To test H3, we ran a three one-way ANOVAs to assess the effects of experimental condition (procedurally just vs. procedurally unjust) on participants’ perceptions of the police. These indicated significant differences between participants in the procedurally just condition and those in the procedurally unjust condition across trust in the police, perceptions of police legitimacy, and identification with the police. Descriptive statistics are presented in Table 5.

**Table 5:** Descriptive statistics for participant perceptions of the police by police behaviour condition

Perceptions of the police	Police behaviour condition				*F*(1, 299)	*η*^2^
	Procedurally just		Procedurally unjust			
	*M*	*SD*	*M*	*SD*		
trust in the police (high = more)	3.70	0.99	3.01	0.91	38.994***	0.12
police legitimacy (high = more)	3.28	0.95	2.80	0.85	16.885***	0.10
police identification (high = more)	3.48	0.86	3.12	0.69	9.376***	0.10

***p < 0.001

Participants exposed to the procedurally just condition demonstrated more positive perceptions of the police than those in the procedurally unjust condition. They exhibited greater trust in the police (*M* = 3.70, SD = 0.99 vs. *M* = 3.01, SD = 0.91; *F*(1, 299) = 38.994, *p* < 0.001, *η*^2^ = 0.12), perceived them as more legitimate (*M* = 3.28, SD = 0.95 vs. *M* = 2.80, SD = 0.85; *F*(1, 299) = 16.885, *p* < 0.001, *η*^2^ = 0.10), and felt a stronger sense of identification with them (*M* = 3.48, SD = 0.86 vs. *M* = 3.12, SD = 0.65; *F*(1, 299) = 9.376, *p* < 0.001, *η*^2^ = 0.10).

### How vicarious experience affects perceptions of the police

We expected our manipulation to influence participants’ perceptions of the police, building on prior research that suggests vicarious experiences of unjust policing can erode trust in police (Ben-Menachem & Torrats-Espinosa, 2024). However, we go further by investigating *why* people respond negatively to procedural injustice and how it damages perceptions of the police [H4].

**Fig. 2:**
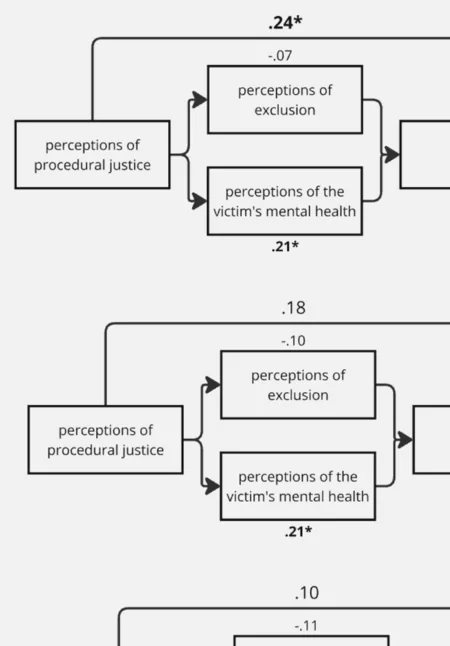
Mediation analysis path models

To test H4, we specified three path models with perceptions of procedural justice as the independent variable, trust in the police (model 1), police legitimacy (model 2), and identification with the police (model 3) as the dependent variables, and perceptions of exclusion and the victim’s mental health as mediators. Exclusion was positioned as a precursor to mental health, reflecting the notion that feelings of exclusion can harm mental health. The models included all possible paths to assess (a) the direct effect of procedural justice on the outcomes (see Table 6) and (b) the extent to which these effects are mediated by exclusion, mental health, or a combination of both. This approach provides insights into whether the recognition of damage to the victim’s mental health has an additional effect on public perceptions of the police (trust, legitimacy, and identification), beyond that of exclusion (and procedural justice) alone. A summary of the mediation analyses is presented in Fig. 2 and Table 7. Model 1 accounted for 45% of the variance in trust (*R*^2^ = 0.45), Model 2 accounted for 35% of the variance in legitimacy (*R*^2^ = 0.35), and Model 3 accounted for 36% of the variance in identification (*R*^2^ = 0.36).

**Table 6:** Bivariate associations between key variables and perceptions of the police

	Trust		Legitimacy		Identification	
	*B*	*SE*	*B*	*SE*	*B*	*SE*
Procedural justice	0.24*	0.09	0.18	0.09	0.09	0.08
Exclusion	0.09	0.09	0.13	0.08	0.14	0.07
Victim’s mental health	0.38*	0.13	0.37*	0.12	0.39**	0.11

**p < 0.001, *p < 0.05

**Table 7:** Mediation analysis summary

Relationship	Total effect	Direct effect	Indirect effect	CI		Conclusion
				Lower	Upper	
PJ > (Exclusion > MH) > Trust	0.39 (*p* < 0.001)	0.24 (*p* = 0.012)	Exclusion: − 0.07 MH: 0.21	− 0.232 0.042	0.069 0.372	Partial mediation via MH
PJ > (Exclusion > MH) > Legitimacy	0.28 (*p* < 0.001)	0.18 (*p* = 0.057)	Exclusion: − 0.10 MH: 0.21	− 0.250 0.054	0.029 0.355	Full mediation via MH
PJ > (Exclusion > MH) > police ID	0.20 (*p* < 0.001)	0.095 (*p* = 0.234)	Exclusion: − 0.11 MH: 0.22	− 0.240 0.085	0.008 0.345	Full mediation via MH

#### Trust in police

Perceptions of procedural justice directly influenced trust in the police (*b* = 0.24, *p* = 0.012). However, perceptions of exclusion did not mediate this relationship (indirect effect =  − 0.07 [− 0.23, 0.07]). In contrast, perceptions of the victim’s mental health partially mediated the relationship between procedural justice and trust (indirect effect = 0.21 [0.04, 0.37]).

#### Police legitimacy

Perceptions of procedural justice had no significant direct effect on police legitimacy when mediators were included (*b* = 0.18, *p* = 0.057). Exclusion did not mediate this relationship (indirect effect =  − 0.10 [− 0.25, 0.03]), but perceptions of the victim’s mental health did (indirect effect = 0.21 [0.05, 0.36]).

#### Identification with police

Procedural justice perceptions did not directly influence identification with the police (*b* = 0.09, *p* = 0.234), nor was exclusion a mediator (indirect effect =  − 0.11 [− 0.24, 0.01]). However, perceptions of the victim’s mental health fully mediated the relationship between procedural justice and identification with the police (indirect effect = 0.22 [0.09, 0.35]).

Across all outcomes—trust, legitimacy, and identification—perceptions of the victim’s mental health emerged as a significant mediator, while perceptions of exclusion did not. In other words, it seems that public recognition of the psychological harm caused by unfair police practices is what drives negative perceptions of the police, rather than the procedurally unjust and exclusionary practices themselves.

### Summary of findings

In line with H1, exposure to procedurally unjust police behaviour led to more negative evaluations of the psychological consequences of police interactions in terms of social exclusion and emotional harm. In agreement with H2, perceptions of social exclusion experienced by the victim partly mediated the relationship between perceptions of procedural justice and perceptions of the psychological harm inflicted on the victim. In line with H3, exposure to a scenario that describes procedurally unjust police behaviour resulted in a loss of trust, legitimacy, and identification with the police. Finally, in agreement with H4, mediation analyses showed that these negative perceptions were primarily driven by the recognition of psychological harm inflicted on the victim, rather than exclusion or the unfairness itself.

The results above show that exclusion mediated the relationship between procedural justice and mental health (PJ → Exclusion → Mental Health). However, when mental health was included as a second mediator in the models predicting overall perceptions of the police (PJ → Exclusion → Mental Health → Perceptions of the police), exclusion no longer mediated the path between procedural justice and the outcome variables. It thus seems that judgements about the exclusionary effect of police injustice have a relatively direct and prominent role in shaping perceptions of the victim’s mental health—perhaps because the latter is inherently linked to feelings of marginalisation and emotional harm. In other words, people ‘see’ that procedural injustice is exclusionary and that this can be bad for the mental health of those who experience it. By contrast, in the model linking procedural justice to perceptions of the police, it is precisely those perceptions of psychological harm that seem to act as the stronger and more immediate mediator. This could suggest that while people recognise that exclusion contributes to psychological harm, they form judgements of the police based on this more immediate harm, i.e. to mental health, rather than the underlying exclusion experienced by the victim.

## Discussion

This study has a number of central findings: (a) individuals recognise the emotional harm caused by police procedural injustice to others and understand that it can contribute to marginalisation and social exclusion; (b) people think procedural injustice is bad for other people’s mental health partly because it contributes to feelings of social exclusion; (c) vicarious exposure to procedurally unjust police behaviour damages general perceptions of the police; (d) trust, legitimacy, and identification with the police are eroded due to an awareness of the psychological harm experienced by others.

These findings shed light on the specific aspects of police behaviour that drive the well-documented negative outcomes associated with exposure to policing (McLeod et al., 2020; Jindal et al., 2022). In particular, the findings suggest that aspects of police behaviour thought to be harmful to well-being of those exposed to policing (directly or vicariously)—particularly procedural injustice—also shape how people perceive the impact of policing on *others*. Emerging research suggests that the psychological impact of policing often hinges on how individuals interpret their experiences with police (Alang et al., 2021). Importantly, our results extend this understanding by showing that these interpretations are not limited to personal experiences; people also recognise that procedural injustice harms the mental health of others and understand that it can contribute to marginalisation and social exclusion. Recognising that procedural injustice contributes to marginalisation and social exclusion offers insight into why it harms mental health. People view procedural injustice as detrimental to others’ mental health in part because it fosters feelings of social exclusion.

This study did not aim to have participants ‘imagine they were in the victim’s shoes’.^3^ Instead, it shed light on the impact of vicarious exposure to policing, which is an important and powerful factor in shaping public responses to, and perceptions of, police. The recognition that procedural injustice can have harmful effects on the mental health of others may be key to understanding why vicarious experiences of unfair policing can significantly impact broader perceptions of police trust and legitimacy. While previous research has shown that witnessing or learning about police mistreatment can damage people’s own views of procedural justice, trust, legitimacy, and other outcomes in much the same way as direct, personal contact does (Foster et al., 2022; Johnson et al., 2017; Ray, 2023; Williamson & Murphy, 2023), our findings add another layer to this understanding. Specifically, when people perceive that unfair treatment by police is not only a violation of justice but also actively harms the well-being of those involved, this awareness may amplify their negative reactions. Indeed, it has been argued that moralistic anger arises not only from personal injustices but also from witnessing harm to others, as individuals feel a strong commitment to their community’s moral norms, and violations of these norms provoke anger and a desire to punish to protect communal integrity (Darley, 2009; Miller, 2001). In this regard, our study is the first to demonstrate that people recognise the psychological harm that police procedural injustice inflicts on others. This further explains why vicarious experiences of unfair policing erode trust, undermine legitimacy, and weaken emotional attachment to the police.

These insights highlight the need for interventions aimed at improving police-public relations to focus not only on promoting fair treatment but also on mitigating the harm caused by unjust practices. Police agencies should consider the psychological mechanisms underlying public perceptions, such as the harm caused by social exclusion and emotional distress, and integrate this understanding into their policies and practices.

One practical implication is the importance of enhancing procedural fairness training for officers. Such training should not only emphasise the need for fair and impartial treatment but also educate officers on the psychological impact of their interactions, particularly the potential for exclusion and harm. For instance, modules could include case studies and role-playing exercises designed to highlight how exclusionary or unjust behaviours affect victims’ mental health and the public’s perceptions of the police. Our findings also support the adoption of trauma-informed policing strategies. These strategies involve training officers to recognise signs of psychological harm and respond in ways that minimise further distress. For example, officers could be trained to communicate empathy, validate victims’ experiences, and provide appropriate referrals to support services, thereby mitigating the negative effects of perceptions of procedural injustice. Beyond individual interactions, broader community-level initiatives could address the residual harm caused by unjust practices. Restorative justice programmes, for instance, could provide victims and community members with a platform to voice their experiences, seek acknowledgment, and engage in dialogue with police.

By addressing both procedural fairness and the resulting psychological impact, police agencies may be able to rebuild trust and strengthen their legitimacy within the communities they serve.

### Limitations and future research

Despite the valuable insights gained from this study, several limitations should be acknowledged. The use of a vignette-based design, while allowing for controlled experimental conditions, simplifies the complexity of real-world police interactions. By focusing primarily on procedural justice, the vignettes may overlook the broader range of behaviours and contextual factors that influence perceptions of police encounters. Additionally, participants are responding to hypothetical scenarios, which may not elicit the same psychological and emotional reactions as actual police interactions. These limitations may affect the generalisability of the findings to real-life contexts, where individuals often experience heightened emotional and psychological responses shaped by a wider array of situational dynamics. Future research should therefore focus on studying real-world vicarious police interactions—such as those witnessed directly or learned about through others—to capture the full emotional and psychological complexity of the public’s reactions to these encounters. This approach would provide a more accurate understanding of how such indirect experiences shape perceptions of procedural justice, trust, and legitimacy.

Another limitation of the current study is the absence of pre- and post-tests for the dependent variables, which restricts our ability to assess changes in participants’ perceptions of the police over time. While the experimental design allows us to infer group differences based on exposure to procedurally just or unjust conditions, a longitudinal design with pre- and post-measures would provide stronger evidence for causal relationships. Specifically, it would enable us to track within-subject changes in perceptions of trust, legitimacy, and identification with the police, as well as perceptions of harm and exclusion. Future research could address this limitation by incorporating pre- and post-tests or employing longitudinal methodologies to more comprehensively evaluate the effects of procedural justice on public perceptions over time.

## Conclusion

This study is the first to show that people recognise the psychological harm procedural injustice causes others, which helps explain why witnessing unfair policing erodes trust, legitimacy, and emotional connection to the police. These effects persisted even after accounting for participants’ socio-economic and demographic characteristics, highlighting the broad relevance of procedural justice in shaping public attitudes. The paper highlights the critical role of psychological harm in driving public responses to unjust policing. Addressing this harm is essential for fostering trust and repairing fractured relationships between police and some of the communities they serve.

## Appendix 1. Vignettes

Please read the story on the next page carefully. You will next be asked a few questions about this story. Click proceed to read the story.

### Procedural justice: Vignette 1

Emily, a 32-year-old woman, was walking home from work late one evening when she was suddenly mugged by a stranger. As she passed a dimly lit alley, a man approached her from behind, grabbed her arm roughly, and demanded her purse. The man had a knife, threatening Emily to comply quickly. Terrified, Emily handed over her purse, which contained her wallet, phone, and keys. The man then pushed her to the ground before fleeing into the night.

Shaken and frightened, she immediately called the police, using the emergency 999 number. Officer Smith and Officer Roberts arrived at the scene about 10 min later. Officer Smith, the first to approach, greeted Emily warmly, introducing himself and Officer Roberts by name. His tone was calm and reassuring. He asked if she was physically okay and whether she needed any medical attention, taking a moment to assess her condition before proceeding with questions.

As Emily began recounting the incident, Officer Smith listened attentively, maintaining eye contact and nodding to show he was engaged. He asked questions in a gentle and respectful manner, giving Emily the time she needed to collect her thoughts and recall the details. When she struggled to remember specific aspects due to the shock, Officer Smith patiently reassured her, saying, ‘Take your time, whatever you can remember will be helpful’.

Throughout the interaction, Officer Smith made sure to explain each step of the process, letting Emily know why certain questions were being asked and how the information would be used to help track down the suspect. When she asked if there were security cameras in the area that could have captured the incident, Officer Smith replied, ‘Yes, we’ll definitely be looking into that as part of the investigation’, emphasising that her case was a priority.

Officer Roberts offered Emily additional support, asking if there was anyone she wanted to contact for assistance or if she needed help getting home. When she expressed concern about not having her keys or phone, Officer Smith offered to have a patrol car drive her home and arranged for a locksmith to meet her there. He provided Emily with his direct contact information, encouraging her to reach out anytime if she had further questions or needed updates on the case.

Before leaving, Officer Smith reiterated that they would do everything they could to find the suspect and recover her belongings. He made sure Emily felt safe and supported before departing, reassuring her that the police were there to help her every step of the way.

### Procedural injustice: Vignette 2

Emily, a 32-year-old woman, was walking home from work late one evening when she was suddenly mugged by a stranger. As she passed a dimly lit alley, a man approached her from behind, grabbed her arm roughly, and demanded her purse. The man had a knife, threatening Emily to comply quickly. Terrified, Emily handed over her purse, which contained her wallet, phone, and keys. The man then pushed her to the ground before fleeing into the night.

Shaken and frightened, she immediately called the police. Officer Smith and Officer Roberts arrived at the scene about 10 min later. Officer Smith greeted Emily, in a neutral and business-like manner. He asked if she was okay, but his demeanour suggested he was more focused on getting through the process than on her well-being.

As Emily began recounting the incident, Officer Smith listened but often seemed distracted, glancing at his phone or the surroundings. He asked questions that felt routine and impersonal, sticking closely to a checklist rather than engaging with Emily’s emotional state. While he wasn’t outright dismissive, there was a noticeable lack of warmth or empathy in his interactions. When Emily hesitated or struggled to remember specific details due to her shock, Officer Smith seemed slightly impatient, prompting her to ‘just do your best’ to recall what she could.

Officer Smith briefly explained that they would file a report and try to locate the suspect, but he didn’t go into detail or provide much reassurance about the process. He didn’t offer any follow-up information, leaving Emily unsure about what would happen next. When she asked if there were security cameras in the area that could have captured the incident, Officer Smith replied, ‘We’ll check on that later’, but his tone suggested it wasn’t a priority.

Emily asked if someone could accompany her home or if there was anything else she should do. Officer Smith, while not rude, simply told her that another unit might pass by later. He handed her a card with a case number but no personal contact information, leaving her uncertain about how to follow up on the case.

Before leaving, Officer Smith briefly mentioned that they would file the report and ‘see what they could do’ to find the suspect and recover her belongings. Without taking the time to ensure she felt secure or supported, Officer Smith simply told her to ‘stay safe’ and then departed, offering little comfort or confidence that the police would be there to help her through the ordeal.

## Appendix 2. Measures

**Table:**

	Measure	Item(s)	*α*
Judgements of procedural justice/injustice displayed by the police	Overall fair treatment	Overall, how fairly do you think Emily was treated?	NA
	Service satisfaction	If you were Emily, would you have been satisfied with the service you received?	NA
	Case satisfaction	If you were Emily, would you have been satisfied with how the police dealt with this case?	NA
	Procedural justice (Thinking about the interaction between Emily and the police, would you agree or disagree that the police…)	Treated Emily with respect and dignity	0.95
		Had the authority to deal with Emily’s problem	
		Were unhelpful (R)	
		Knew what they were talking about	
		Tried as hard to help Emily as they would anyone else	
		Were impartial, i.e. acted in a fair and neutral manner	
		Did not understand Emily’s problem (R)	
		Gave Emily the opportunity to express her views before decisions were made	
		Listened to Emily before making decisions	
Perceptions of exclusion experienced by the victim during the interaction	Exclusion (To what extent do you think Emily’s interaction with the police would have made her feel…)	Excluded from society	0.86
		Less important than other people in her community	
		Like she doesn’t belong in society	
		Like society is on the side of victims like her (R)	
Perceptions of the victim's mental health outcomes as a result of the interaction	Do you think that Emily will have left the interaction with the police feeling…	Stressed (R)	0.97
		Anxious (R)	
		Traumatised (R)	
		Resilient	
		Empowered	
		Positively emotionally impacted	
		Negatively emotionally impacted (R)	
		Fearful (R)	
		Depressed (R)	
		Angry (R)	
		Helpless (R)	
		Validated	
		Hopeful	
		Isolated (R)	
		Safe	
		Trusting of authority	
		Confused (R)	
		Understood	
		Reassured	
	If you were in Emily's position, experiencing the situation as described in the story, how do you think you would feel after the interaction with the police?	Stressed (R)	0.97
		Anxious (R)	
		Traumatised (R)	
		Resilient	
		Empowered	
		Positively emotionally impacted	
		Negatively emotionally impacted (R)	
		Fearful (R)	
		Depressed (R)	
		Angry (R)	
		Helpless (R)	
		Validated	
		Hopeful	
		Isolated (R)	
		Safe	
		Trusting of authority	
		Confused (R)	
		Understood	
		Reassured	
Perceptions of the police	Trust in the police (To what extent do you agree or disagree that…)	The police explain their decisions to the people they deal with	0.83
		The police would treat you with respect if you had contact with them for any reason	
	Police legitimacy (To what extent do you agree or disagree that…)	The police can be trusted to make the right decisions	0.90
		The police usually act in ways that are consistent with my own ideas about what is right and wrong	
		The police stand up for moral values that are important for people like me	
		It is my moral duty to back the decisions made by the police because the police are legitimate authorities	
		It is my moral duty to support the decisions of police officers, even if I disagree with them	
		It is my moral duty to do what the police tell me even if I don’t understand or agree with the reasons	
	Identification with the police (Please indicate to what extent you agree or disagree with the statements below.)	I identify with the police	0.82
		I feel a sense of solidarity with the police	
		I feel similar to the police	
		I see myself as an honest, law-abiding citizen	
		It is important to me that others see me as an honest, law-abiding citizen	
Demographics^*^	Age	How old are you? e.g. 18–24	NA
	Gender	Are you: male; female; non-binary/third gender; prefer not to say	NA
	Sexual orientation	Please select the option that best describes your sexual orientation	NA
	Ethnicity	To which of these ethnic groups do you consider you belong?	NA
Socio-economic variables^*^	Education	What is the highest level of education you have completed?	NA
	Employment status	Are you e.g. employed	NA
	Disability	Do you have any long-standing illness, disability or infirmity?	NA
	Residential area	Which of the following best describes your residential area?	NA
	Deprivation	Which of the descriptions below comes closest to how you feel about your household’s income nowadays?	NA
	Financial difficulty	If for some reason you were in serious financial difficulties and had to borrow money to make ends meet, from the bank or family, how difficult or easy would that be?	NA
	Victimisation	Have you been a victim of crime in the past 2 years?	NA
Qualitative question	Comments	If you have any further comments or feedback, we welcome your input here	NA

*Response categories have been omitted from the table for simplicity of presentation

## Footnotes

1 We use the terms ‘procedural injustice’ and related phrases such as ‘procedurally unjust police behaviour’ interchangeably. This approach aligns with common practices in the procedural justice literature, where such terms are used contextually to capture both the theoretical concept and specific instances of police behaviour. Where applicable, we have ensured clarity by using the term most appropriate to the context. Moreover, both ‘procedural justice’ and ‘procedural injustice’ can be appropriate, depending on context.

2 Most participants did not provide additional comments, but among the 30 who did, several highlighted narrative inconsistencies, particularly around Emily’s actions after her phone was stolen. Some found it challenging to separate their emotional responses to the crime from their views on police behaviour, expressing ambivalence. A few praised the study’s design, though some had concerns about question phrasing. Others shared personal opinions on policing, discussing issues like bias, the need for reform, and the impact of police behaviour on different social groups, reflecting broader societal concerns and calls for improved police vetting processes.

3 Although participants’ ratings of how they believed Emily felt after the interaction with the police and how they imagined they would have felt in her position were strongly correlated.
